# Analysis of climate variability, trends, and prediction in the most active parts of the Lake Chad basin, Africa

**DOI:** 10.1038/s41598-019-42811-9

**Published:** 2019-04-19

**Authors:** Rashid Mahmood, Shaofeng Jia, Wenbin Zhu

**Affiliations:** 1 Key Laboratory of Water Cycle & Related Land Surface Processes/Institute of Geographic Science and Natural Resources Research, Chinese Academy of Sciences, Beijing, 100101 China; 2 Qinghai Key Laboratory of Basin Water Cycle and Ecology, Qinghai Institute of Water Resources and Hydropower, Xining, China; 3 School of Geographical Sciences, Qinghai Normal University, Xining, China

**Keywords:** Climate change, Projection and prediction

## Abstract

An understanding of climate variability, trends, and prediction for better water resource management and planning in a basin is very important. Since the water resources of the Lake Chad basin (LCB) are highly vulnerable to changing climate, in the present study, a combination of trend analysis methods was used to examine the climate variability and trends for the period of 1951–2015 using observed and Climate Research Unit (CRU) data, and a combination of spectral analysis techniques was used for the prediction of temperature and precipitation using CRU data. Eighty-four percent of the temperature time series indicated extremely strong signals of increasing trends (α = 0.001) and 25–38% of the precipitation time series indicated strong decreasing trends (α = 0.05). Temperature is expected to increase and precipitation is expected to decrease in the future. However, surprisingly, in some regions located in the South, the temperature was predicted to decrease slightly in 2021–2030 relative to 2006–2015. This decrease might occur because these regions are highly protected natural resource areas and forests are frequently present. On the whole, the temperature was predicted to increase by 0.65–1.6 °C and precipitation was predicted to decrease by 13–11% in the next two decades (i.e., 2016–2025 and 2026–2035) relative to 1961–1990. Periodic analysis showed a 20- to 25-year cycle in precipitation in all basins and a 40- to 45-year cycle in temperature but only in the Chari-Logone basin.

## Introduction

The changing climate, global warming, and global energy imbalance are considered the main consequences of the dramatic increase in the concentration of greenhouse gases due to human activities, especially industrialization, the burning of fossil fuel, and land use/land cover changes^1–3^. One noticeable example of changing climate is an increase in the global average temperature by 0.74 °C ± 0.18 °C in only the last 100 years (1906–2005)^4^. This global warming has been accelerating and intensifying the water cycle of the world, which can result in more violent storms, floods, and droughts^5^. The intensified water cycle can influence every aspect of human activities, such as human health, water energy exploitation, ecosystems, food security, and industrial and municipal water supplies^3^. However, changes in the water cycle vary from region to region throughout the world^6^. Although climate change is one of the largest threats to the world, the poor regions, especially in Africa, have been suffering the most because these areas are least equipped to cope with climate change impacts^7–9^. According to Serdeczny *et al*.^10^, Africa has been identified as the most vulnerable continent to climate change impacts, and specifically, the water resources of Africa are highly vulnerable to climate change. One noticeable example is the water resources of the Lake Chad basin (LCB), where inflow to Lake Chad (LC) has diminished by 70–80%^11^, and the surface area of the lake has been reduced to approximately 2,000 km^2^ from 25,000 km^2^ (approximately 90%) over the last 40 years^12^, and even in the 1980s, it shrank to 300 km^2^^12^. In addition, the lake was divided into the southern and northern pools in 1975 because of a devastating drought over the Sahel belt during the 1970s. Since then, the northern pool has hardly been flooded^13^. Furthermore, due to the shallow water level, LC is highly sensitive to changing climate, especially changes in precipitation and temperature^13^.

This occurrence shows that water resources of the LCB are highly vulnerable to rapidly changing global and regional climate, especially changes in temperature and precipitation, which are considered the most important elements in climate description^14^. The changes in these two variable can easily alter the hydrological cycle and environmental processes^15^. A good description of temperature and precipitation trends and variability is essential for many studies related to hydrology, climatology, and agriculture. Long-term trend analysis in temperature and precipitation is highly important for rainfed agricultural regions, where farmland is mainly reliant on precipitation, and for irrigated regions, where both temperature and precipitation can affect irrigation scheduling^15^. Trend analysis of climate variables is the central process in assessing the state of the climate of a region and provides an overall estimate about the variations in the climate variables^16^. Therefore, a good knowledge of trends in temperature and precipitation is very important for better water resource management in a basin, water demand and supply, agricultural water use and regulation, and even for better planning in a region.

Because knowledge of climate variability and trends is important for many aspects, the accurate forecast/prediction of climate variables is also equally important for the policymakers, planners, and other people working on water resource management and on mitigation and adaptation measures to cope with climate change. In addition, such predictions are also important for managing water supply and demand, mitigating flood and drought, maintaining reservoir water levels, planning and preparing for disasters, reducing the uncertainty by providing information on the future availability of water, improving water resources allocation, etc.^15,17–19^.

For the assessment of climate variability and trends, different parametric (e.g., T-test, F-test, and linear regression) and nonparametric (e.g., Mann–Kendall test, Kruskal–Wallis test, and Sen’s slope estimator) methods have been reported in the literature and are reviewed in detail in these studies, such as Esterby^20^, Zang *et al*.^21^, and Sonali and Nagesh^22^. Although parametric methods are more powerful, their applications are restricted to normally distributed time series. Since most of the climatic time series, specifically precipitation, do not fulfill normality requirement, nonparametric methods are most frequently applied in trend analysis, as in Burn *et al*.^23^, Fu *et al*.^24^, *Oyerinde et al*.^25^, Tekleab *et al*.^26^ and Wang *et al*.^27^. In addition, the nonparametric methods are considered more robust than parametric methods against the outliers in a time series^21,22^.

On the other hand, for climate prediction, time series modeling is one of the major tools used when predicting short- and long-term changes in a climatic time series^17,28^. Time series analyses are usually used for monitoring, forecasting, and feedback by fitting a suitable model to a long time series. These time series analyses are widely used scientific methods in different field of studies, such as regional development and planning, business management, weather forecasting, market potential prediction, hydrological forecasting, environmental pollution control, astronomy, and oceanography^29,30^. Different kinds of techniques, such as moving average (MA), autoregressive (AR), autoregressive and moving average (ARMA), integrated ARMA (ARIMA), exponential smoothing (ES), seasonal decomposition, neural network, and spectral analysis, have been developed to analyze patterns in time series and for forecasting^31^. However, time series, specifically precipitation, are mostly considered difficult to predict because these consist of different components, such as trends, periodic patters, cyclic behavior, and white noise (random component), which limit the application of simple and widely used forecasting methods as mentioned above. Thus, the time series having complex cyclic behavior of different frequencies are better addressed with spectral analysis^32^. The spectral analysis is mostly used to study the physical processes in geophysics^33^, to study the stars in astronomy^34^ and to predict the weather in meteorology^35^.

Different studies have been reported in the literature to explore the climate variability and general trends in or around the LCB, such as Adeyeri *et al*.^36^, Funk *et al*.^37^, Nkiaka *et al*.^38^ and Okonkwo *et al*.^39^. Adeyeri *et al*.^36^ conducted trend analysis in the Komadugu-Yobe basin located in the northwest of the LCB using observed and gridded reanalysis precipitation for the period of 1979–2015, and Nkiaka *et al*.^38^ used observed precipitation for 1950–2000 in the Logone River basin located in the south of the LCB. Okonkwo *et al*.^39^ applied linear trends in CRU precipitation for rainy season (June–September) for the period of 1970–2010. However, they used only mean values from the southern LCB and northern LCB. Funk *et al*.^37^ explored trends in observed temperature and precipitation over Chad for the period 1900–2009 but only for the rainy season (June–September). However, these studies covered the LCB only partially, and none of the studies have been reported to cover the whole conventional basin, which is the most active part in the LCB and contributes entirely to LC; in addition, they did not even cover the Chari-Logone River basin, the river that provides more than 90% water to the lake. Moreover, most studies focused on precipitation and only the rainy season.

Many studies such as Buontempo^40^, Francois *et al*.^41^, Sarr^42^, Sultan *et al*.^43^, Sylla *et al*.^44^, Sylla *et al*.^45^, and Vizy *et al*.^46^ reported projections for the temperature and precipitation in the 21^st^ century around the LCB using RCMs and/or GCMs under RCPs. However, with the best of our knowledge, no studies have been reported in the LCB for predicting temperature and precipitation for the near future using time series analysis, which is also a reasonably good, fast technique for use in predicting meteorological variables for the near future. It also requires less computational requirements and is simple to use.

**Figure 1:**
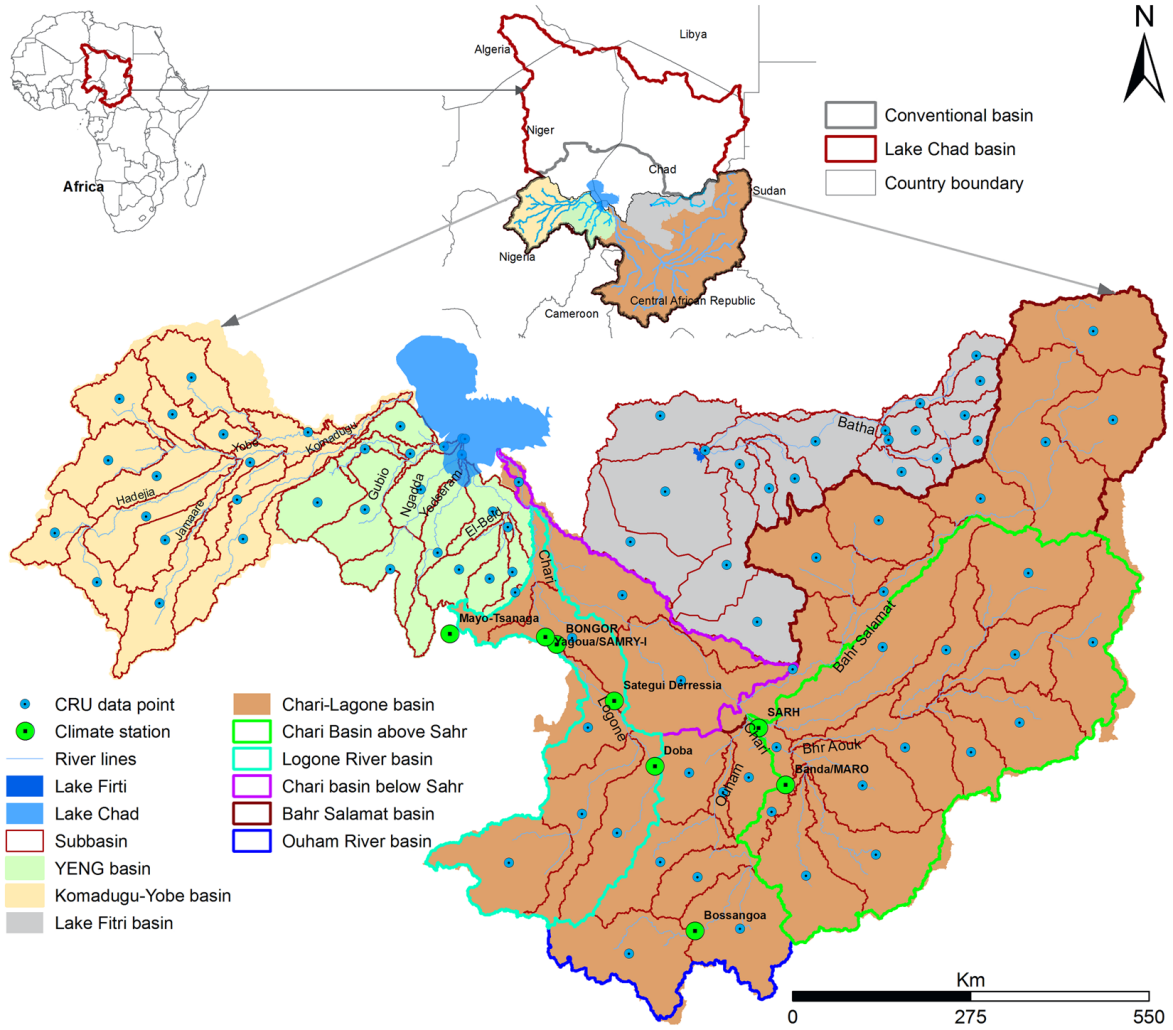
Location map showing the extracted CRU climate data points, observed meteorological stations, and subbasins in the study area, the Lake Chad basin. CRU data points are obtained by taking the mean of all grids inside a subbasin. YENG refers to *Yedseram, El-Beid, Nagada and Gubio River*.

Thus, the study had the following as the main objectives: (1) to assess the climate variability and climatic trends in the LCB by a combination of linear regression, polynomial model, Sen’s slope, and Mann-Kendal test of the annual and seasonal temperature and precipitation, which is essential for water resource management, and (2) to apply a combination of spectral analysis and harmonic regression to predict both variables and changes were assessed in these variables relative to the present condition, which is of great importance for water resource planning under anticipated climate change and for the mitigation of flood and droughts in the basin. In addition, periodic analysis was also performed to explore the cyclic behavior of temperature and precipitation. These analyses were performed in the most active parts of the LCB, encompassing the Chari-Logone, Komadugu-Yobe, Yedseram, El-Beid, Ngadda, Gubio, and Batha River basins (Fig. 1) for the period of 1951–2015. Temperature and precipitation were predicted for the next two decades (i.e., 2016–2025 and 2026–2035), and decadal changes were examined relative to the baseline period (1961–1990).

This study is a part of an ongoing project “Feasibility of Inter Basin Water Transfer Project” of Power-China International Limited, aiming to restore the rapidly shrinking LC to its required ecological level by transferring water from the Congo River. The present study will help in water resources management and planning as well as in mitigating floods and droughts in the basin.

## Study Area, Data and Methods

### Study area

The Lake Chad basin (LCB) is the world’s biggest endorheic and transboundary drainage basin, covering an area of 2.5 × 10^6^ km², which is approximately 8% of Africa^11,12^. It is situated between 5.2 and 25.3°N latitude and between 6.9 and 24.5°E longitude and straddles the borders of seven countries: Algeria, Cameroon, Central African Republic, Chad, Libya, Niger, Nigeria, and Sudan, as shown in Fig. 1. This basin is a part of the Sahel belt and has a semiarid climate^39^. The whole basin mainly contributes water to Lake Chad (LC), which is one of the biggest lakes in the world. It is a vital source of fresh water for pastoral land, agricultural land, and fishing^47^. Among these uses, agriculture is the major user of its water resources, which provides livelihood to approximately 60% of the population in the LCB^48^. The LCB receives an average annual rainfall of 415 mm, ranging from 20–150 mm in the northern parts (e.g., Algeria) to 1200–1600 mm in the southwestern parts of the basin (e.g., Central African Republic)^49^. The average annual temperature varies between 35 °C and 40 °C. The basin is hot and dry during March to June, wet and hot during June to October, and dry and relatively cool from November to February^50^.

The present study was conducted in the most active parts of the LCB, which include the Chari-Logone, Komadugu-Yobe, Yedseram, El-Beid, Ngadda, Gubio, and Batha River basins (Fig. 1) and cover approximately 36% (911202 km^2^) of the LCB^8,50^. All these rivers contribute directly to LC except the Batha River, which provides water only to Lake Fitri (Fig. 1). Among them, the Chari-Logone River is the main river system, which enters to the southern pool of the lake and contributes more than 90% to the total water of the lake. The Komadugu-Yobe River (KYR) is the second main river system, which enters to the northern pool of the lake and contributes 2–5% water to the lake^12,51^. In addition, the YENG (Yedseram, El-Beid, Ngadda, and Gubio Rivers) flows in the southwest of LC and contributes even less than 2%^50^.

### Data description

Monthly precipitation data from 8 meteorological stations were obtained from the Lake Chad Basin Commission (LCBC) for the period of 1950–2013, as shown in Fig. 1; these stations did not adequately cover the large study area.

Recently, leading scientific research centers from around the world have created globally interpolated gridded datasets for understanding and predicting weather, water, and climate of the world, such as Climate Research Unit (CRU) dataset, Global Precipitation Climatology Center (GPCC), CICS High-resolution Optimally interpolated Microwave Precipitation from Satellite (CHOMPS), National Center for Environmental Protection (NCEP), and the Tropical Rainfall Measurement Mission (TRMM). These datasets have been created by observed, satellite, reanalysis, and combination of satellite and observed data.

In the present study, due to a scarcity of observed data, monthly mean temperature and precipitation were obtained from the latest version CRU-TS4.00 for the period of 1951–2015 because this dataset has been used widely in different studies in Africa^52–54^ and in different studies for the evaluation of global climate models, regional climate modes, and hydrological models, as in Chiyuan *et al*.^55^, McMahon *et al*.^56^, and Smiatek *et al*.^57^. In addition, CRU data were evaluated with *in situ* observed data using different statistical indicators in Mahmood and Jia (2018), which showed that CRU data can be used with high confidence. This is a high-resolution (0.5° × 0.5°) monthly climatic dataset, including precipitation, max temperature, min temperature, mean temperature, wet days, potential evapotranspiration, diurnal temperature range, vapor pressure, cloud cover, and frost-day frequency for the period of 1901 to 2016^58^.

In the present study, the whole study area was divided into 84 subbasins using digital elevation model (DEM) to take care of spatial variability, as shown in Fig. 1, and the CRU data was extracted for each subbasin by taking the average of CRU-grids covering that subbasin. Therefore, the CRU extracted data for each basin and observed data were used in trend analysis, and only the CRU data was used in the time series analysis for predicting temperature and precipitation.

### Trend analysis

In the present study, Mann-Kendal (MK)^59,60^, a widely used nonparametric method, was applied to identify statistically significant trends in temperature and precipitation for the period of 1951–2015, using both observed and CRU data. For a time series *x*_1,_*x*_2,_*x*_3,_…, *x*_*n*_, with *n* > 10, the MK test statistic (S), the variance of MK test statistic V(S), and the associated standard normal test statistic (Z) are calculated as below^15,16,61^:

$${\rm{Z}}=[\begin{array}{cc}\frac{{\rm{S}}-1}{\sqrt{{\rm{V}}({\rm{S}})}} & {\rm{for}}\,{\rm{S}} > 0\\ 0 & {\rm{for}}\,{\rm{S}}=0\\ \frac{{\rm{S}}+1}{\sqrt{{\rm{V}}({\rm{S}})}} & {\rm{for}}\,{\rm{S}} < 0\end{array}]$$

$${\rm{S}}=\sum _{{\rm{i}}=1}^{{\rm{n}}-1}\,\sum _{{\rm{j}}={\rm{i}}+1}^{{\rm{n}}}\,{\rm{sgn}}({{\rm{x}}}_{{\rm{j}}}-{{\rm{x}}}_{{\rm{i}}})$$

$${\rm{sgn}}({{\rm{x}}}_{{\rm{j}}}-{{\rm{x}}}_{{\rm{i}}})=[\begin{array}{cc}1 & \mathrm{for}\,\,({{\rm{x}}}_{{\rm{j}}}-{{\rm{x}}}_{{\rm{i}}}) > 0\\ 0 & \mathrm{for}\,\,({{\rm{x}}}_{{\rm{j}}}-{{\rm{x}}}_{{\rm{i}}})=0\\ -1 & \mathrm{for}\,\,({{\rm{x}}}_{{\rm{j}}}-{{\rm{x}}}_{{\rm{i}}}) < 0\end{array}]$$

$${\rm{V}}({\rm{S}})=\frac{1}{18}[{\rm{n}}({\rm{n}}-1)(2{\rm{n}}+5)-\sum _{{\rm{p}}=1}^{{\rm{q}}}\,{{\rm{t}}}_{{\rm{p}}}({{\rm{t}}}_{{\rm{p}}}-1)(2{{\rm{t}}}_{{\rm{p}}}+5)]$$

where *q* represents total number of tied groups. A set of the same values in a dataset is referred to as a tied group. Each tied group is denoted by *t*_*p*_. The positive values of *Z* indicate upward (increasing) trends in time series, and the negative values show downward (decreasing) trends. Trends are then tested against some critical values (*Z*_1−*α*_) to show that either they are statistically significant or not. For example, if |*Z|* > *Z*_*1*−*α*_, (e.g., *Z*_*1*−*α*_ at α = 0.05); the null hypothesis of no-trend is rejected, and alternative hypothesis of significant trend is accepted.

To quantify the magnitude of detected trends, a frequently used nonparametric method, the Sen’s slope method^62^, was applied in the present study, as in Khattak *et al*.^6^, Burn *et al*.^47^, Kumar *et al*.^63^, and Mahmood and JIA^16^. This method is robust against outliers in a time series. Sen’s slope (*SS*) is calculated as below:

$$SS=median[\frac{{x}_{j}-{x}_{i}}{j-i}]\,{\rm{for}}\,{\rm{all}}\,i < j$$

where *xi* is the value of data at time step *i and x*_*j*_ at time step *j*. Before application of the MK test, the time series must be free of serial correlation, which can mislead the actual result of the trends. To remove serial correlation from a time series, Trend-Free Pre-Whitening (TFPW)^64^ was used in the present study. This method has been used for different studies, as in Burn *et al*.^23^, Khattak *et al*.^6^, and Kumar *et al*.^63^, to remove serial correlation.

In the present study, the MK and SS methods were applied on 84 time series for each variable (temperature and precipitation) to explore trends and to calculate the magnitude of trends, after application of the TFPW method. The trends were detected for annual CRU temperature as well as for annual and seasonal (dry and wet) CRU and observed precipitation in the study area for the period of 1951–2015 at four significance levels (i.e., α = 0.10, 0.05, 0.01, and 0.001). Two seasons, the wet season and the dry season, are present in the all basins but have different time intervals, as described in Komble *et al*.^50^. According to Komble *et al*.^50^, precipitation for the wet season was calculated from May to October in the CLRB, from June to September in the KYRB and YENG, and from July to September in the LFB, and precipitation for the dry season was obtained from the rest of the months.

### Time series analysis

In the present study, a combination of harmonic regression and spectral analysis was used to better deal with time series, such as precipitation, which has complex periodic and cyclic behavior. Therefore, the periodic component (sinusoidal component) in the time series (*Y*_*t*_) can be attained by the following model, as in Grzesica and Więcek^32^ and Hintze^65^:

$${{\rm{Y}}}_{{\rm{t}}}={\rm{\mu }}+{\rm{R}}\,\cos \,[{\rm{ft}}+{\rm{d}}]+{\in }_{{\rm{t}}}$$

where *µ* is the mean of the time series, R is the amplitude (height or magnitude) of the wave, *f* shows the frequency of periodic variation, the wavelength of each wave can be obtained by dividing 2π by *f*, and *d* is the horizontal offset or phase. Changes in *d* shifts the beginning of the cycle.*∈*_*t*_ is a white noise or random error in the time series, which causes difficulty to determine the periodic pattern in the series. *t* is the time period of observation (*t* = 1, 2, 3…, N), where N is the total number of observations.

In practice, capturing the variation of a time series requires the sum of several different sinusoidal waves of different wavelengths (or frequencies). Further, since temperature and precipitation time series have strong trends, especially in temperature, eventually the time series are either removed by differencing (lag 1) to make it stationary, which is the requirement of this method, or the trend term (*mt*) can be added to the harmonic regression model to forecast the time series properly. By adding the trend term, as in Hintze^65^, in the harmonic regression, the final model can be written in the form of sum of *k* frequencies required to fit the model into a time series.

$${Y}_{t}=\mu +mt+\sum _{j=1}^{k}\,{a}_{j}\,\cos ({f}_{j}t)+\sum _{j=1}^{k}\,{b}_{j}sin\,({f}_{j}t)+{\epsilon }_{t}$$

where *a*_*j*_ (j = 1, 2, 3…k) and *b*_*j*_ are regression coefficients to be estimated in this harmonic regression model. In this method, the central process is to determine an appropriate set of frequencies to regenerate the time series, which can explain the cyclic behavior of the time series. This is mostly done by inspecting the periodogram, which is obtained by spectral analysis (Fourier analysis). A periodogram is a plot to identify a set of frequencies (wavelengths) in the time series. In the present study, we used NCSS statistical software for spectral analysis and harmonic regression for forecasting of temperature and precipitation. From these periodogram plots, peak values are selected and used in the harmonic regression model. However, these values are just estimates; the most suitable frequencies are selected by trial and error method. More detail about spectral analysis and harmonic regression is given in^29,32,65–67^.

In this study, time series analysis was performed on 84 time series (37 for the CLRB, 15 for the KYRB, 17 for the YENG, and 15 for the LFB) of each climatic variable (i.e., temperature and precipitation) extracted from the CRU datasets. For detailed analysis, the CLRB was divided into 5 subbasins (Fig. 1): that is, the Chari River basin above Sarh (CRBAS), the Chari River basin below Sarh (CRBBS), the Bahr Salamat basin (BSB), the Ouham River basin (ORB), and the Logone River basin (LRB). The other three basins (i.e., KYRB, YENG, and LFB) were divided into the northern, southern, eastern, and western regions.

## Results

**Figure 2:**
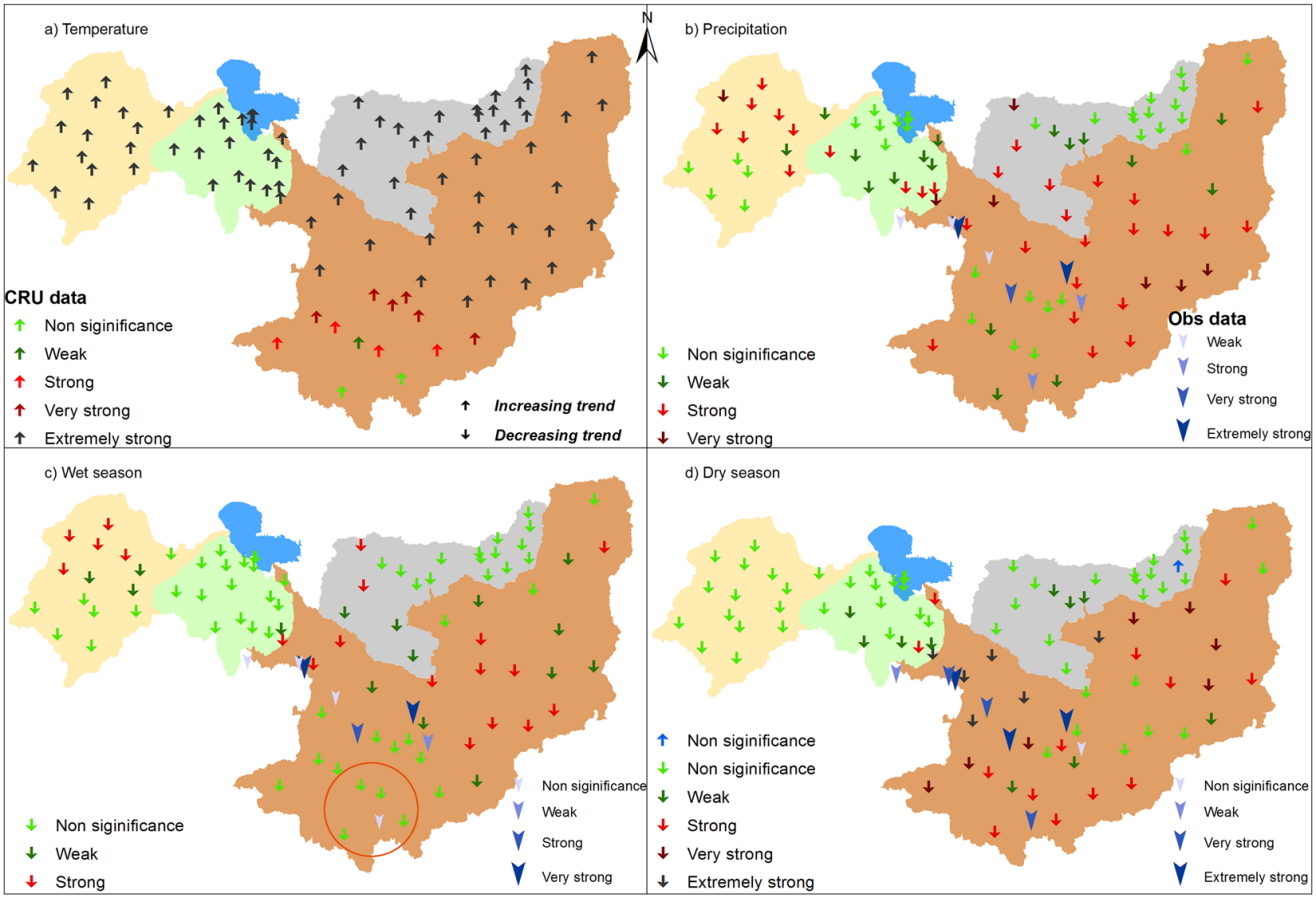
Spatial distribution of trends (**a**) annual temperature, (**b**) annual precipitation, (**c**) wet season precipitation, and (**d**) dry season precipitation in the study area, calculated from CRU and observed data for the period of 1951–2015. Trend strength is categorized as (1) trends at α = 0.1 (weak signal of trend), (2) trends at α = 0.05 (strong), (3) trends at α = 0.01 (very strong), and (4) trends at α = 0.001 (extremely strong).

**Figure 3:**
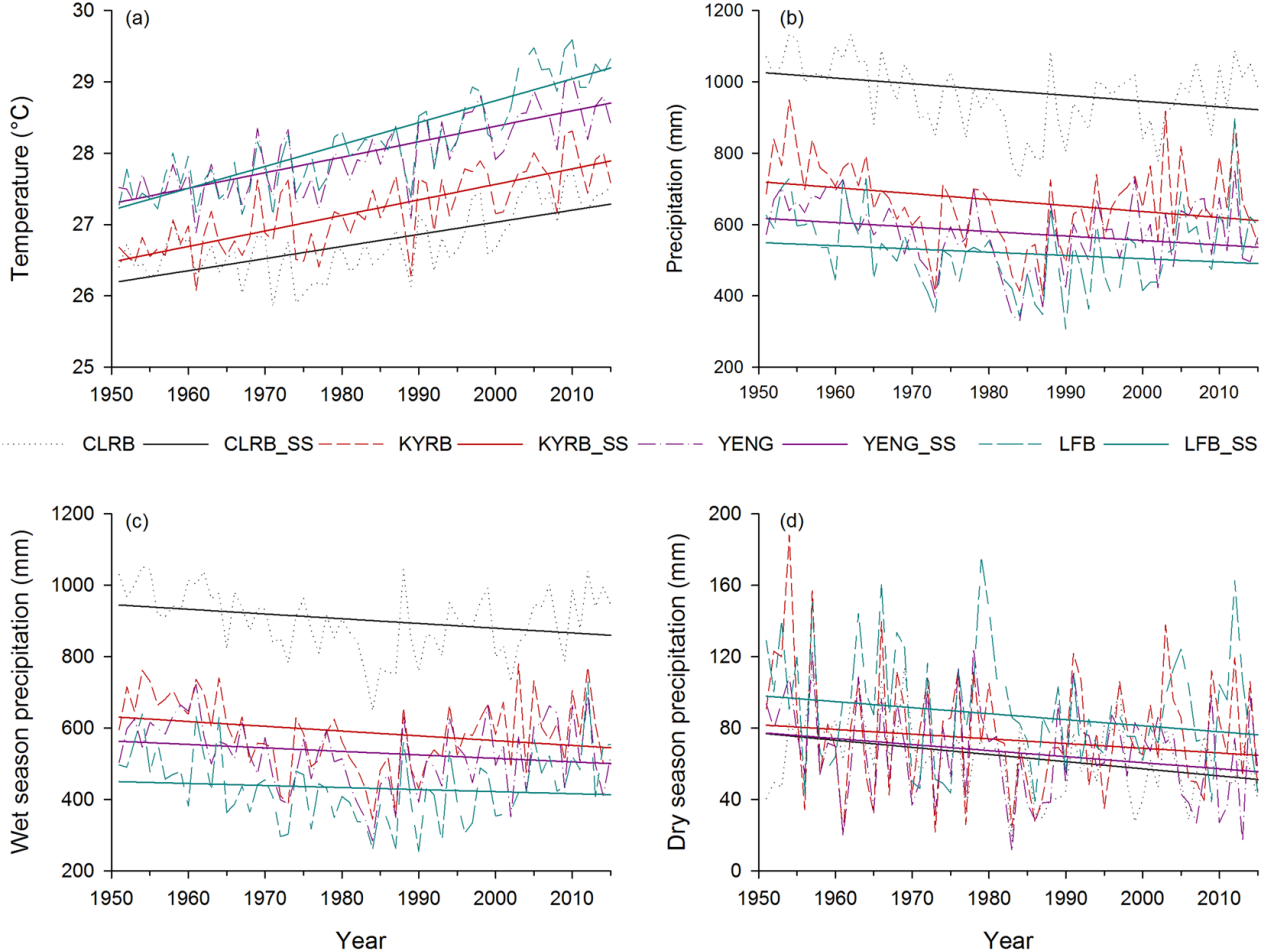
Temporal trends of (**a**) annual temperature, (**b**) annual precipitation, (**c**) wet season precipitation, and (**d**) dry season precipitation in the study area. Here, CLRB (Chari-Logone River basin), KYRB (Komadugu-Yobe River basin), YENG (Yedseram, El-Beid, Nagada, and Gubio River basins), LFB (Lake Fitri basin), and SS (Sen’s slope estimator).

**Figure 4:**
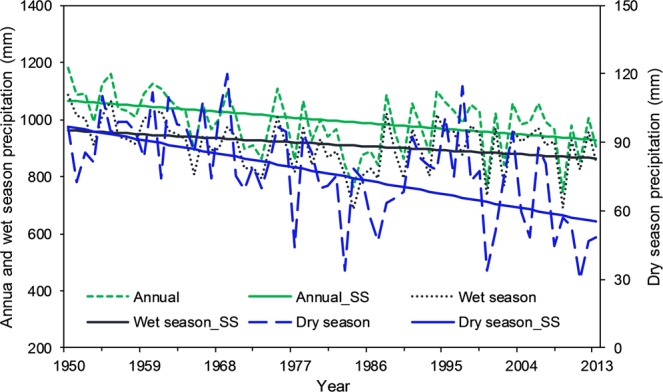
Trend lines for observed mean annual and seasonal precipitation for the period of 1950–2013 in the Chari-Logone River basin (SS Sen’s slope estimator).

Figure 2 shows the temperature and precipitation trends calculated by MK test for the period of 1951–2015 in the study area (i.e., the CLRB, KYRB, YENG, and LFB), and the magnitude of trend lines calculated by Sen’s slope method are displayed in Figs 3 and 4. The strength of the trend signals was divided into four categories on the basis of significance level: (1) trends at α = 0.1 (weak signal of trend), (2) trends at α = 0.05 (strong), (3) trends at α = 0.01 (very strong), and (4) trends at α = 0.001 (extremely strong), as shown in Fig. 2.

### Temperature trends

Figure 2a shows spatially distributed annual temperature trends obtained in the study area and displays upward trends in all time series. Among them, 84% explored extremely strong increasing trends. However, all time series in the KYB, YENG, and LFB had extremely significant rising trends. In the CLRB, approximately 96% of the time series showed increasing trends. Among them, most of the time series (approximately 64%) displayed extremely strong increasing trends, and only 2 time series, situated in the south of the CLRB, displayed nonsignificant trends. Figure 2a also shows stronger effects of global warming in the northern areas relative to the southern regions. Figure 3a shows temperature trend lines obtained with Sen’s slope method in the CLRB, KYRB, YENG and LFB, representing significant upward trends. The trend line of the LFB before 1960s was lower than YENG’s trend line, but then it increased at a faster rate than YENG’s rate of increase. This suggests that increasing rate in temperature is higher in the northeastern parts of the basin than southwestern regions. In the whole study area, an average rate of increasing temperature was estimated to be 0.022 °C yr^−1^ (1.43 °C per 65 years), with 0.017 °C yr^−1^, 0.021 °C yr^−1^, 0.022 °C yr^−1^, and 0.029 °C yr^−1^ in the CLRB, KYRB, YENG, and LFB, respectively.

### Precipitation trends

#### Annual trends using CRU data

Figure 2b displays spatially distributed annual precipitation trends in the study area for the period of 1951–2015, showing downward trends on all time series. Among them, 38% of total time series disclosed strong and 9% very strong evidence of decreasing precipitation over the whole study area. Nonetheless, 53% of the time series showed no or weak decreasing signals, and none of the time series showed extremely strong deceasing trends. Fifty-seven percent (21 time series out of 37) of the time series in the CLRB and 53% (8 out of 15) in the KYB were explored to have strong to very strong decrease trends. In the YENG and LFB, most of the time series (70–75%) displayed no or weak decreasing signals. Figure 3b shows precipitation trends lines calculated by Sen’s slope (SS) method in the CLRB, KYRB, YENG. All trend lines showed downward trends in all four basins, although the rate of decrease was not as strong as the temperature’s rate of increase. A decreasing rate of 1.7 mm yr^−1^, 1.5 mm yr^−1^, 1.22 mm yr^−1^, and 1.20 mm yr^−1^ were explored in the CLRB, KYB, YENG, and LFB, respectively, with an average decrease of 1.4 (14 mm decade^−1^) in the whole study area. This means that, in the next 100 years, the basin will receive approximately 20–25% less precipitation relative to the current conditions if the same rate of decrease will continue in the basin. These results show that the study area is not much affected in the case of decreasing precipitation in comparison to the increasing temperature because, after the 1980s, a gradual increase in precipitation was observed (Fig. 3b). However, a little change in precipitation may have greater impacts on water resources.

#### Seasonal trends using CRU data

Figure 2c,d show the trends for wet and dry seasons distributed spatially in the study area, for the period of 1951–2015. In the wet season, only 25% of the time series were observed to have strong decreasing trends, and 75% disclosed nonsignificant decreasing trends in the whole study area. Thirty-two percent of the time series in the CLRB, 33% in the KYRB, and 17% in the LFB had strong decreasing trends, whereas none of the time series in the YENG showed this trend. In the dry season, extremely strong decreasing trends were observed on 8% of the time series, very strong on 9%, and strong on 17% in the whole study area. However, 66% showed none significant decreasing signals. Most of the strong to extremely strong decreasing signals (approximately 68%) were explored in the CLRB, and almost none of the time series showed significant decreasing in other three basins, except 1 time series in the YENG basin with strong signal. In the whole CB, only 1 time series located in the LFB was found to have an increasing, though nonsignificant, signal.

The trend lines in all 4 basins for the wet and dry seasons are shown in Fig. 3c,d, indicating downward trend lines in all basins, though not highly significant. An average decreasing rate of precipitation in the wet season was estimated as 1.07 mm yr^−1^ (11 mm decade^−1^) in the whole basin, with the highest decline in the CLRB (1.4 mm yr^−1^) and the lowest in the LFB (0.8 mm yr^−1^), which are, nonetheless, less than the annual precipitation rates. In the case of dry season, an average decreasing rate was 0.3 mm yr^−1^ (3 mm decade^−1^), with the maximum (0.4 mm yr^−1^) in the CLRB (like in wet season) and minimum (0.2 mm yr^−1^) in the KYRB. In the next 100 years, if these decreasing rates prevail in the basin, then 17% of the precipitation is expected to decrease in the wet season and 36% in the dry season relative to the current conditions. Although the decreasing rate in the wet season was higher than in the dry season, the percentage decrease was higher in the dry season because the dry season receives much lower annual precipitation (70–100 mm yr^−1^) than the wet season (500–1100 mm yr^−1^). Therefore, a little change in dry season will have a greater effect.

#### Annual and seasonal trends using observed data

Figure 2b–d show annual and seasonal precipitation trends spatially distributed in the basin, for the period of 1951–2013, while covering only a very small part of the study area and mostly located in the CLRB. In annual precipitation, 5 of the time series (62%) displayed strong decreasing signals; among them 2 showed extremely strong, 1 very strong, and 2 strong, and the rest (3 time series) showed no significant trends. In the wet season, 37% (3 gauges) of the time series showed strong decreasing signals. However, in the case of the dry season, 75% of the gauges (6 gauges) had extremely strong signals of decreasing. The results obtained from observed stations were stronger but closely matched with the results obtained from CRU measuring points. For example, a nonsignificant trend was shown by observed stations in the wet season (Fig. 2c, circle) at the bottom of the CLRB, and the same trends were detected on the CRU measuring point near the observed precipitation station.

Figure 4 shows the trend lines of annual as well as wet and dry seasonal precipitation for 1950–2013, indicating a clear picture of decreasing precipitation in both annual and seasonal cases. This trend also showed that the dry season’s precipitation decreased at a faster rate than in the annual and wet season, which is the same as for the CRU. The rates of decrease in precipitation in the annual, wet season, and dry season were estimated to be 2.4 mm yr^−1^, 1.7 mm yr^−1^, and 0.62 mm yr^−1^, respectively. Fourteen percent, 11%, and 43% precipitation decreased in the annual, wet and dry cases, respectively, since 1951. According to these rates, in the next 100 years, the basin will receive 22% less annual precipitation relative to the current condition, 17% less precipitation in the wet season, and 66% less precipitation in the dry season.

### Predictions of temperature and precipitation

#### Evaluation of the fitted model

**Figure 5:**
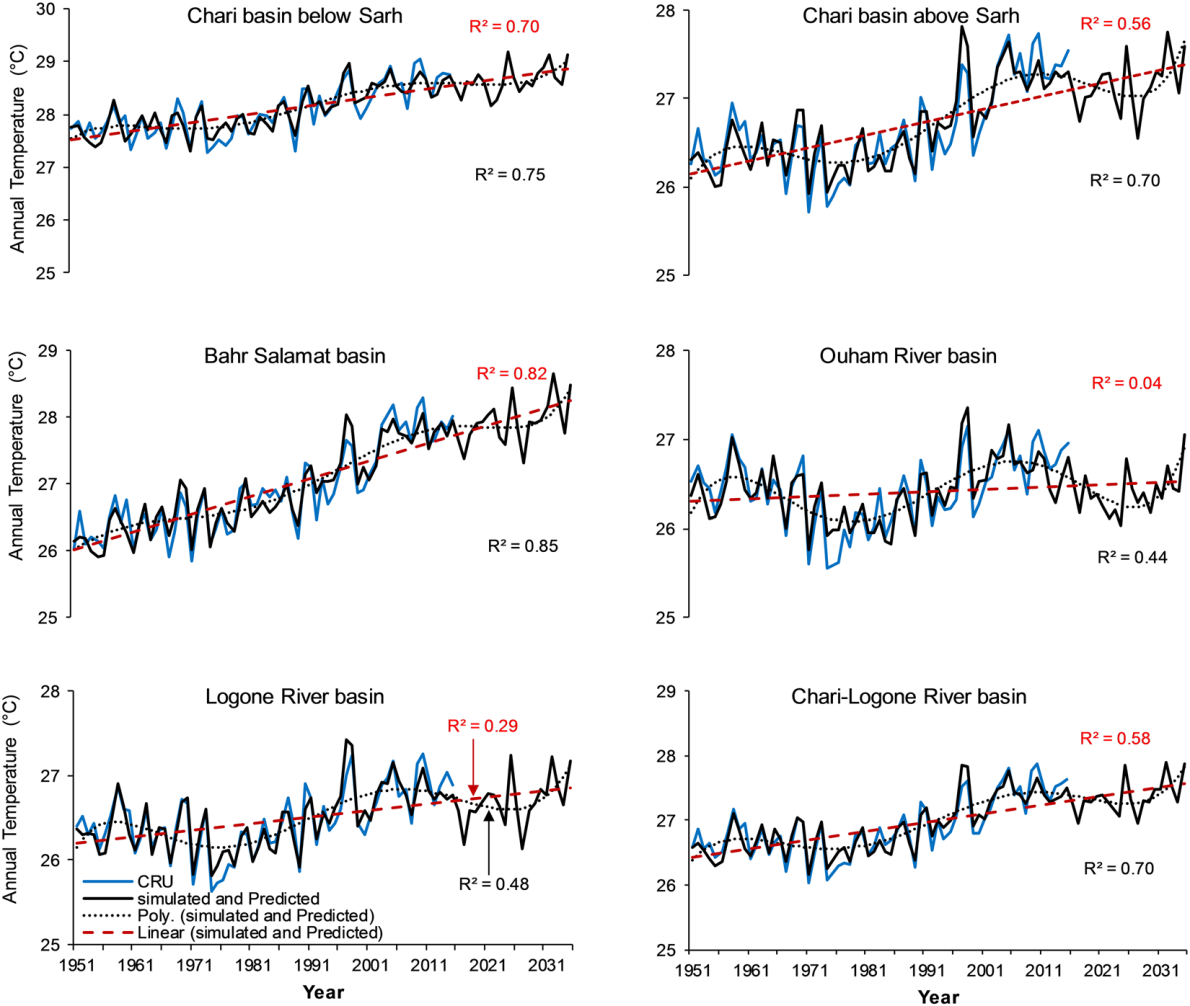
Simulated (1951–2015) and predicted (2016–2035) temperature time series against the CRU temperature in the subbasins of Chari-Logone River basin. *R*^2^ (red) regression coefficient and *R*^2^ (black) polynomial regression coefficient.

**Figure 6:**
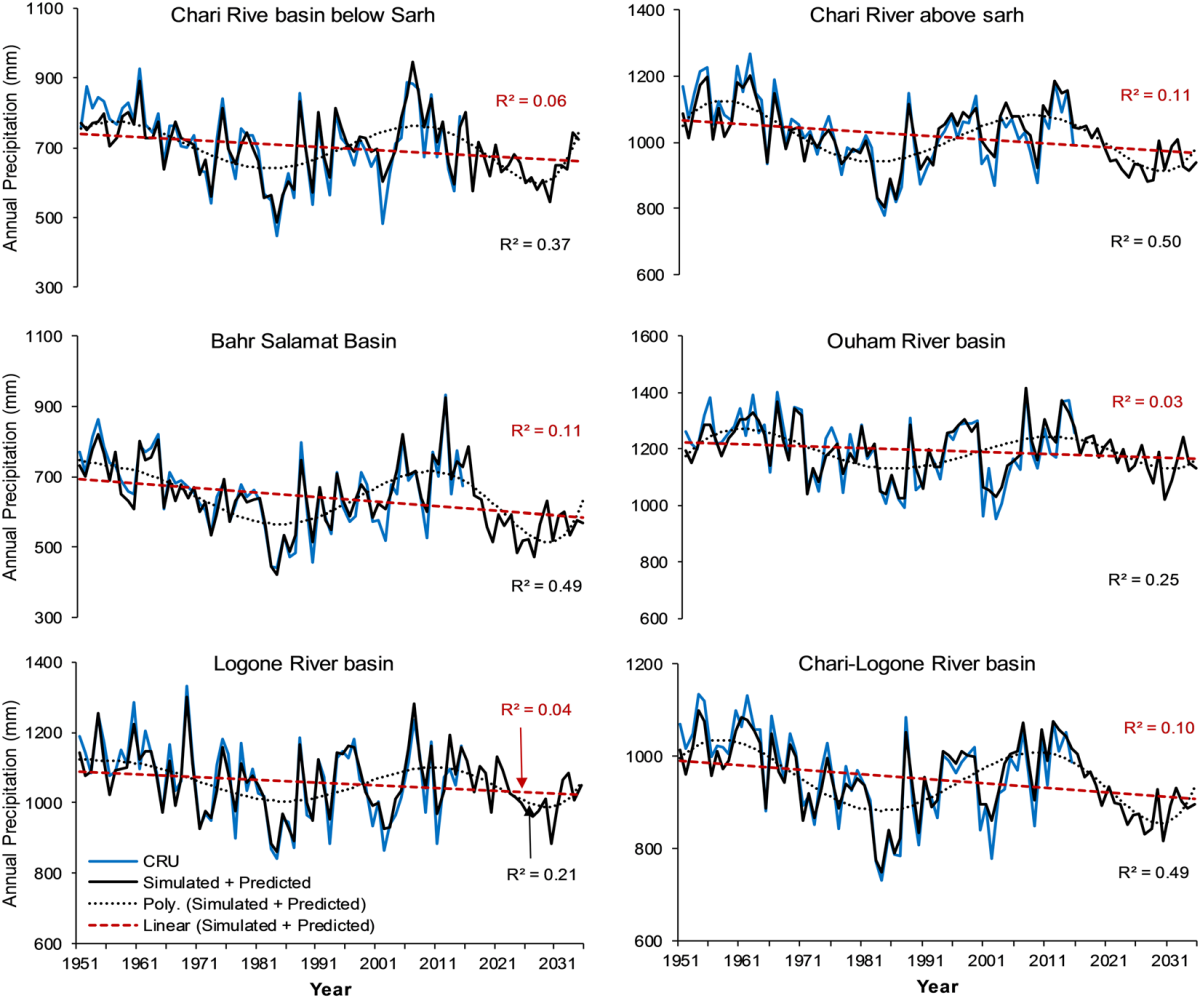
Simulated (1951–2015) and predicted (2016–2035) precipitation time series against the CRU in the subbasins of Chari-Logone River basin. *R*^2^ (red) linear regression coefficient and *R*^2^ (black) polynomial regression coefficient.

**Figure 7:**
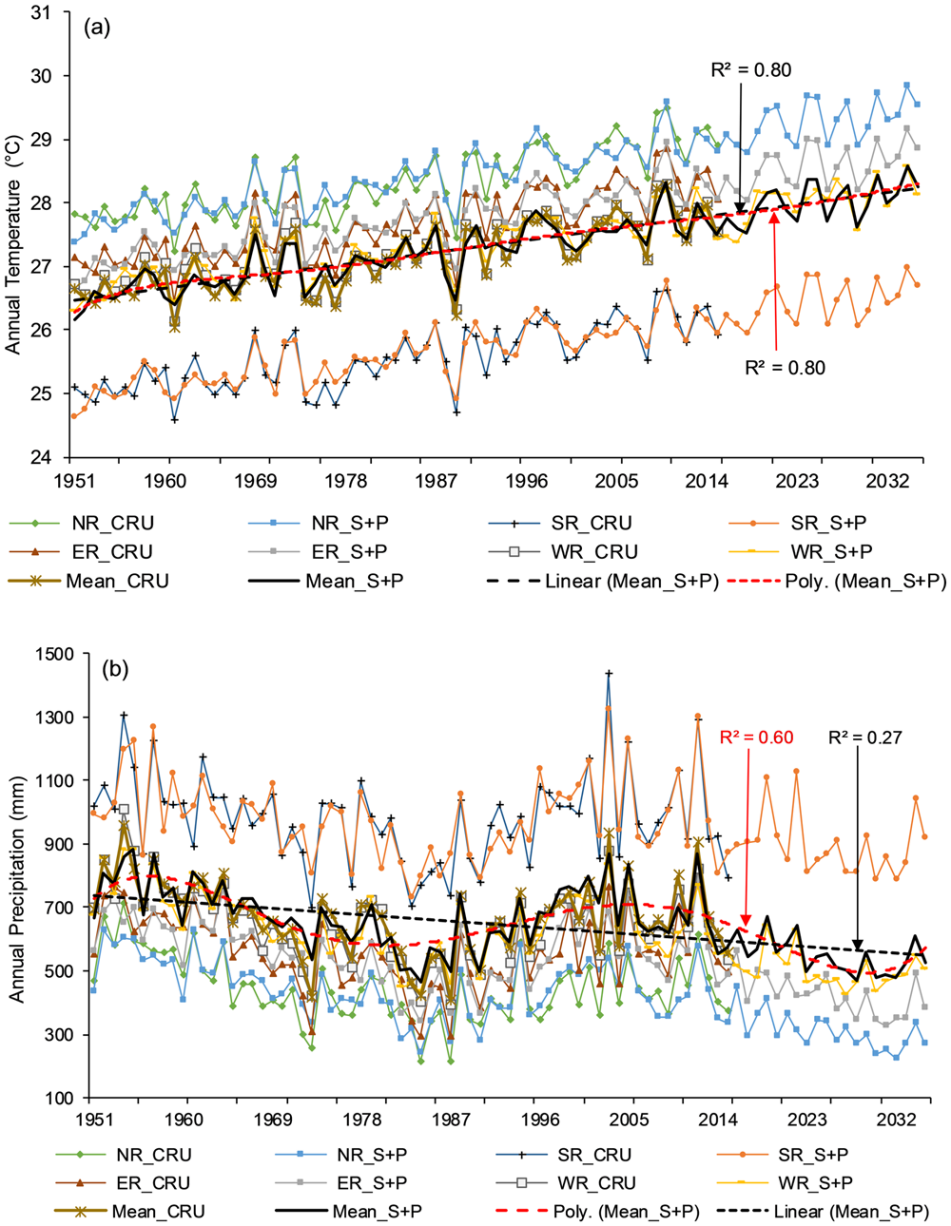
Simulated (1951–2015) and predicted (2016–2035) (**a**) temperature and (**b**) precipitation against the CRU in the Komadugu-Yobe basin. Herein, NR (Northern region), SR (Southern region), ER (Eastern region), WR (Western region), S + P (Simulated + Predicted), *R*^2^ (red) linear regression coefficient, and *R*^2^ (black) polynomial regression coefficient.

**Figure 8:**
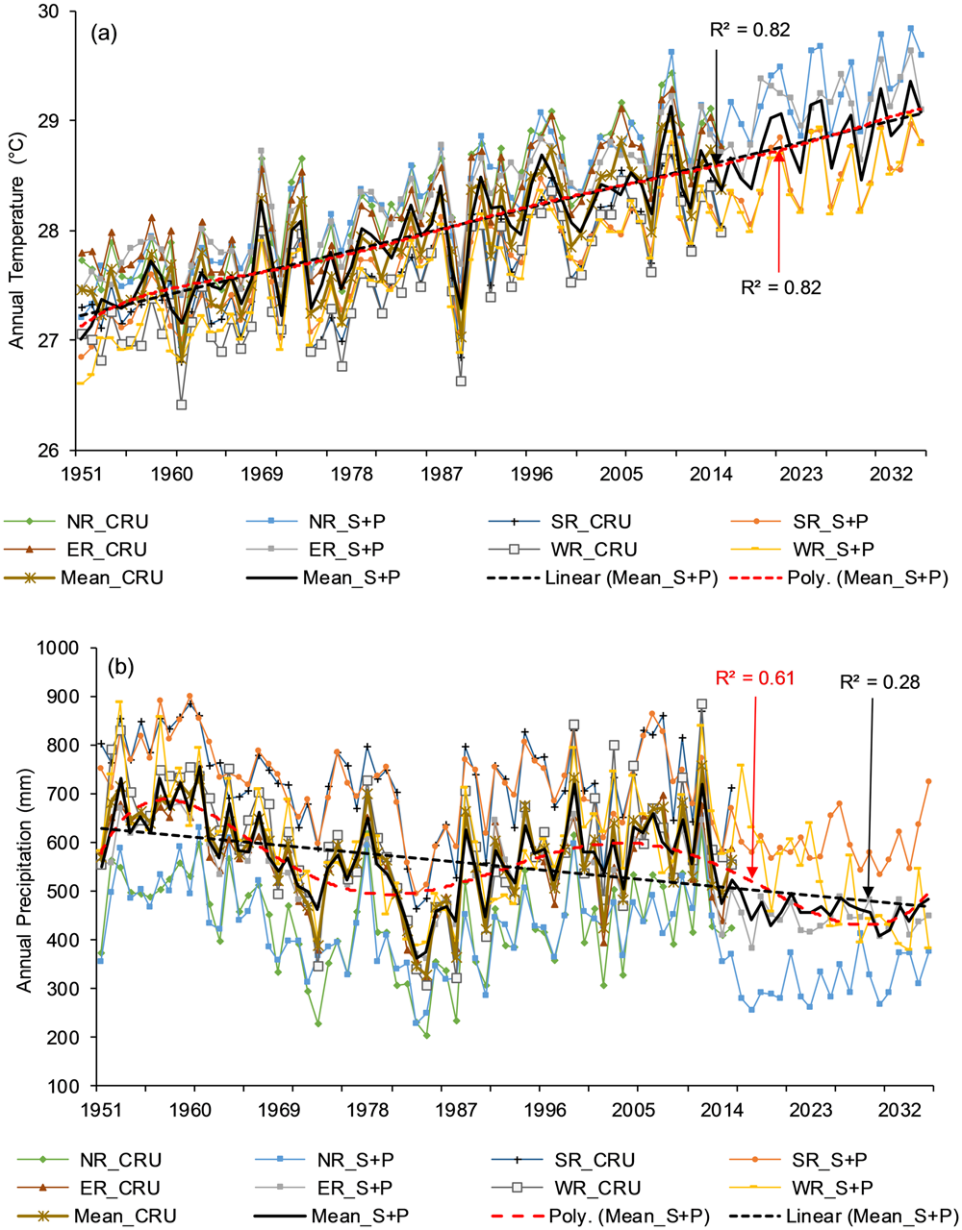
Caption of Fig. 8 for the YENG basin.

**Figure 9:**
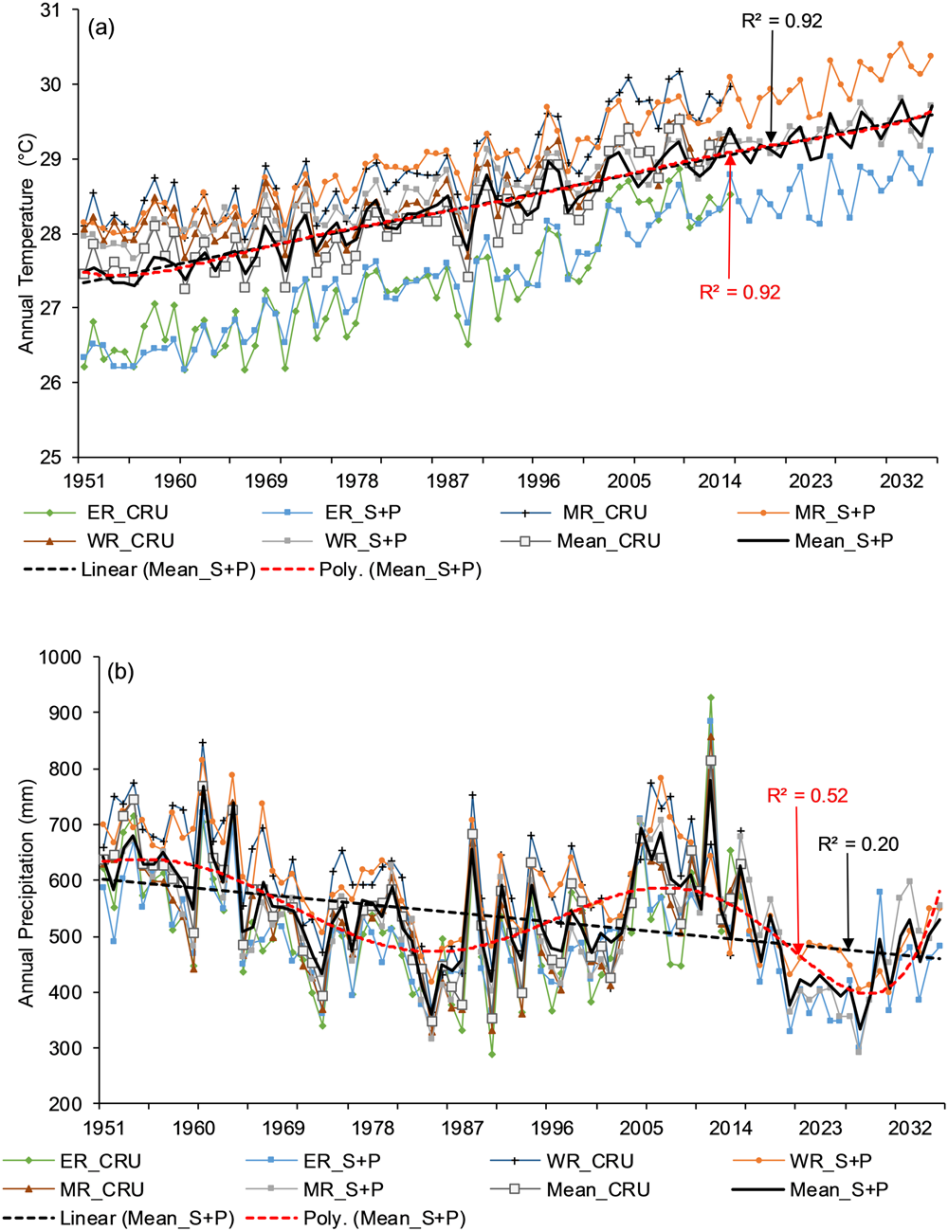
Simulated (1951–2015) and predicted (2016–2035) (**a**) temperature and (**b**) precipitation against the CRU in the Komadugu-Yobe basin. Herein, ER (Eastern region), MR (Middle region), WR (Western region), S + P (Simulated + Predicted), linear regression coefficient R2 (red), and polynomial regression coefficient R2 (black).

For the evaluation of the fitted model, coefficient of determination (R^2^), root mean square error (RMSE), error in CRU and simulated means (E_m_), and error in CRU and simulated standard deviations (E_std_) were calculated from CRU and simulated data (i.e., temperature and precipitation) for the period of 1951–2015, for each time series in the study area. The average values of these indicators for the whole study area are described in Table 1. R^2^ values ranged from 0.83 to 0.89 for temperature and from 0.76 to 0.89 for precipitation, and the RMSE values were 0.15–0.23 °C and 29–55 mm yr^−1^, respectively, for temperature and precipitation. E_m_ values were extremely small, 0.0001–0.0013 °C (temperature) and 0.003–0.032% (precipitation). Although the model mostly underestimated in the case of E_std_ values, these were reasonably well inside the acceptable range, with 0.003–0.032% for temperature and 10–13% for precipitation. For more illustration, the simulated temperature and precipitation of each subbasin of the CLRB, KYRB, YENG, and LFB were plotted against the CRU to check how accurately the model follows the variations of CRU, as shown in Figs 5–9. These plots showed that the fitted model captured the variations of the CRU temperature and precipitation well in each subbasin. These indicators and plots showed that the model performed extremely well to regenerate a time series during the evaluation period 1951–2015 in all basins, and thus, has capability to predict temperature and precipitation.

**Table 1:** Evaluation of the fitted model to temperature and precipitation for the period of 1951–2015 in the Chari-Logone, Komadugu-Yobe, YENG, and Lake Fitri basins.

Temperature (°C)	Chari-Logone basin	Komadugu-Yobe basin	YENG basin	Lake Fitri basin
R^2^	0.89	0.84	0.85	0.83
RMSE	0.15	0.19	0.19	0.23
E_m_	0.0013	0.0002	0.0013	0.0001
E_std_	0.034	−0.042	−0.038	−0.058
**Precipitation (mm)**				
R^2^	0.80	0.76	0.84	0.89
RMSE	55	53	37	29
E_m_	−0.003	−0.025	0.026	−0.032
E_std_	−10.3	−11.9	−12.5	−13.1

E_m_ *error in CRU and simulated means*, E_std_ *error in CRU and simulated standard deviations*, R^2^ *coefficient of determination*, RMSE root mean square error.

After a successful evaluation, the fitted model was used to generate temperature and precipitation for the next 20 (values) years (2016–2035), as shown in Figs 5–9. The generated data were divided into 2016–2025 (First Decade—FD) and 2026–2035 (Second Decade—SD), and decadal changes were assessed with respect to the baseline period (1961–1990). Linear regression and polynomial (Hexic degree) trend lines were also fitted to simulate (1951–2015) and predict (2016–2035) data (Figs 5–9) to explore the linear trends and periodic cycles in the time series. We also estimated changes in 2021–2030 (2020 s) relative to 2006–2015 (the present decade—PD) because the troughs of the cycles occurred during the 2020 s (Figs 5–9), and the cyclic behavior showed that there could be more interesting results in the 2020 s relative to the present decade, especially in the CLRB.

#### Chari-Logone River basin

Table 2 describes the changes in temperature and precipitation in the FD and SD with respect to the baseline period and changes in the 2020 s relative to the present decade, in the CLRB. In the FD and SD, an increase in temperature of 0.12–1.28 °C and 0.34–1.63 °C, respectively, was predicted in the basin. The lowest increase was noticed in the ORB located in the South and the highest in the BSB located in the northeast in both decades. In the whole CLRB, the temperature was predicted to increase by 0.65 °C and 0.93 °C in the FD and SD, respectively, indicating clear signals of warming in the basin in the near future. Nonetheless, in the 2020 s, the LRB, CRBAS, and ORB displayed decreases in temperature relative to the PD, with −0.13, −0.16, and −0.33 °C, respectively, showing a cooler climate in the 2020 s relative to the present decade.

**Table 2:** Temperature and precipitation changes in 2016–2025 and 2026–2035 relative to the baseline period (1961–1990) and in the 2020s relative to the present decade (2006–2015) in the subbasins of the Chari-Logone River basin.

Temperature (°C)	Logone basin	Chari basin above Sarh	Bahr Salamat basin	Ouham basin	Chari basin below Sarh	Chari-Logone basin
Absolute CRU 1961–1990	26.2	26.3	26.6	26.2	27.8	26.6
FD (2016–2025)	0.39	0.70	1.28	0.12	0.77	0.65
SD (2026–2035	0.65	1.02	1.63	0.34	1.03	0.93
Absolute CRU 2006–2015	26.8	27.3	27.8	26.6	28.6	27.4
2020 s (2021–2030)	−0.13	−0.16	0.10	−0.33	0.03	−0.10
**Precipitation**						
Absolute CRU 1961–1990 (mm)	1044	1006	628	1184	685	938
FD (2016–2025) (%)	−0.2	−4.9	−12.6	−0.1	−3.5	−3.9
SD (2026–2035) (%)	−3.4	−4.5	−7.8	−5.4	−3.1	−4.9
Absolute CRU 2006–2015 (mm)	1120	1078	725	1273	776	1021
2020 s (2021–2030) (%)	−10.1	−14.2	−24.7	−9.9	−19.1	−14.4

FD *First decade*, SD *Second decade*, CRU *Climate Research Unit*.

In the case of precipitation, all the subbasins showed negative (decreasing) changes in the next two decades relative to the baseline, with 3.9% decrease in the FD and 4.9% in the SD in the whole CLRB. The highest decrease of 12.6% (FD) and 7.8% (SD) were estimated in the BSB, and the lowest decrease of 0.1% (FD) and 3.1% (SD) were observed in the ORB and CRBBS, respectively. Some regions (i.e., BSB and CRBBS, CRBAS) showed a greater increase in precipitation in the SD than FD. However, the decrease in precipitation in the 2020 s was estimated to be more severe relative to the PD, with an average decrease of 14.4% (10–25%) in the whole CLRB.

Figures 5 and 6 shows linear regression and polynomial trend lines (Hexic degree) fitted to simulated (1951–2015) and predicted (2016–2035) temperature and precipitation in the subbasins of the CLRB. All linear regression trend lines showed increasing temperature, with strong regression coefficient (*R*^2^) except in the ORB and LRB. These lines also showed that increasing rate was faster in the northern and eastern regions (e.g., BSB, and CRBAS) than the southwestern regions such as LRB and ORB. It was also observed that *R*^2^ were higher with lines having faster rates of increase. By polynomial lines and spectral analysis, cyclic behavior was explored in temperature, repeating every 45–49 years (Fig. 5) in the different subbasins; however, these showed an upward trend. *R*^2^ for polynomial lines were even stronger than the linear regression lines (Fig. 5). As mentioned above, these polynomial lines also showed that 2020 s will be cooler than the PD, especially in the CRBAS, ORB, and LRB.

In the case of precipitation (Fig. 6), linear regression lines illustrated decreasing trends; however, *R*^2^ were not as strong as temperature. These lines also showed that rates of decrease in precipitation were faster in the regions located in the northeast of the basin (e.g., BSB, CRBBS) than the regions in the south (e.g., ORB). By polynomial lines and spectral analysis, we explored a cyclic behavior of wet and dry periods in precipitation repeating every 20–25 years (Fig. 6), with a downward direction. The repeating cycle showed that next decades will be drier than the present decade.

#### Komadugu-Yobe River basin

The changes in temperature and precipitation for the FD and SD relative to baseline and for the 2020 s relative to the PD are described in Table 3. Temperature was predicted to rise by 0.9–1.1 °C and 1.2–1.3 °C in the FD and SD, respectively, in different regions of the basin. Unlike the CLRB, temperature was estimated to increase in the 2020 s, with an overall increase of 0.31 °C. In contrast, precipitation was predicted to reduce by 1–21% and 5–29% in the FD and SD, respectively, in different parts of the basin. The highest increase of 1.1 °C (1.3 °C) in temperature and the highest decline of 21% (29%) in precipitation were explored in the northern region in the FD (SD). In the 2020 s, decreasing precipitation ranged between 10% and 25% relative to the PD, slightly higher than the SD. In the whole KYRB, the temperature will increase by 1.0 °C in the FD and 1.2 °C, in the SD, and precipitation will decrease by 11% and 15% in the FD and SD, respectively.

**Table 3:** Temperature and precipitation changes in 2016–2025 and 2026–2035 relative to the baseline period (1961–1990) and in the 2020s relative to the present decade (2006–2015) in the subbasins of the Komadugu-Yobe basin.

Temperature (°C)	Northern Region	Southern Region	Eastern Region	Western Region	Basin mean
Absolute CRU 1961–1990	28.10	25.40	27.40	27.00	26.92
FD (2016–2025)	1.10	1.00	1.00	0.90	1.00
SD (2026–2035)	1.30	1.20	1.30	1.20	1.20
Absolute CRU 2006–2015	28.94	26.13	28.27	27.74	27.71
2020 s (2021–2030)	0.32	0.31	0.28	0.32	0.31
Precipitation					
Absolute CRU 1961–1990 (mm)	402	923	515	599	618
FD (2016–2025) (%)	−21	−1	−15	−16	−11
SD (2026–2035) (%)	−29	−5	−22	−18	−16
Absolute CRU 2006–2015 (mm)	407	971	553	607	652
2020 s (2021–2030) (%)	−25	−10	−25	−20	−18

FD *First decade*, SD *Second decade*, CRU *Climate Research Unit*.

The annual simulated and predicted temperature and precipitation are plotted in Fig. 7a,b, and regression and polynomial lines were fitted to the mean data of the KYRB. Regression lines showed clear increasing trend in temperature and decreasing in precipitation, and polynomial lines fitted to temperature displayed same trend such as regression (Fig. 7a). However, from the polynomial line and spectral analysis, we explored a repeating cycle (20–25 years) of wet and dry period in precipitation (Fig. 7b), similar to the LCRB. Linear and polynomial models were better (with high values of R^2^) fitted to temperature and precipitation in the KYRB than the CLRB. Unlike the CLRB, the polynomial model (Hexic order) did not showed any clear repeating cycle of hot and cold periods in the KYRB.

#### YENG basin

Table 4 describes the temperature and precipitation changes in the FD and SD with respect to the baseline and in the 2020 s relative to the PD in different regions of the YENG basin. Temperature was predicted to increase by 1.0–1.2 °C and 1.2–1.4 °C in the FD and SD, respectively. In the 2020 s, temperature will be 0.33 °C higher than in the present decade. On the other hand, precipitation was predicted to decrease by 4–24% in the FD and 13–23% in the SD, and in the 2020 s, the reduction in precipitation was even higher, ranging between 19% and 31% relative to the PD. The most affected region by increase in temperature and decrease in precipitation was the northern region in the basin, same as the CLRB and KYRB. In the whole basin, the temperature will increase by 1.1 °C in the FD and 1.3 °C in the SD, and precipitation will decrease by 14% in the FD and 16% in the SD. The simulated/predicted annual temperature and precipitation are shown in Fig. 8(a,b), along with the linear and polynomial lines. Strong linear and polynomial trends (R^2^ = 0.81) in temperature displayed a definite increase but no periodic behavior such as that observed in the CLRB (Fig. 8a). Both linear and polynomial models showed downward lines in the case of precipitation, and a similar cyclic behavior of the wet and dry period moving downward (Fig. 8b) was observed in the basin by spectral and polynomial analysis.

**Table 4:** Temperature and precipitation changes in 2016–2025 and 2026–2035 relative to the baseline period (1961–1990) and in the 2020 s relative to the present decade (2006–2015) in the subbasins of the YENG basin.

Temperature (°C)	Northern Region	Southern Region	Eastern Region	Western Region	Basin Mean
Absolute CRU 1961–1990	28.00	27.40	28.00	27.30	27.67
FD (2016–2025)	1.20	1.00	1.10	1.10	1.10
SD (2026–2035)	1.40	1.20	1.30	1.30	1.30
Absolute CRU 2006–2015	28.94	28.18	28.80	28.20	28.52
2020 s (2021–2030)	0.31	0.35	0.33	0.30	0.33
**Precipitation**					
Absolute CRU 1961–1990 (mm)	394	687	524	555	536
FD (2016–2025) (%)	−23	−13	−15	−4	−14
SD (2026–2035) (%)	−15	−14	−16	−23	−16
Absolute CRU 2006–2015 (mm)	462	732	572	633	594
2020s (2021–2030) (%)	−31	−19	−22	−20	−22

FD *First decade*, SD *Second decade*, CRU *Climate Research Unit*.

#### Lake Fitri basin

Table 5 describes the temperature and precipitation changes in the FD (SD) and 2020 s relative to the baseline period and the PD, respectively, in different regions of the LFB. In the whole basin, temperature was predicted to increase by 1.24 °C in the FD and 1.6 °C in the SD. Unlike the CLRB, the basin will be warmer in the 2020 s, with 0.34 °C higher temperature than the present decade. On the other hand, precipitation was expected to decrease by 21% and 8% in the FD and SD, respectively, in the whole basin. The highest decrease of 21% and 16% was estimated in the middle and western parts of the basin in the FD and SD, respectively. Unlike the other three basins, the reduction of precipitation was lower in the SD than in the FD. This means precipitation will start increasing after the FD of drought. In the 2020 s, the decline in precipitation ranged between 29% and 37% relative to the PD, which was much higher than other basins. The eastern parts of the basin will be warmer than the other parts. Figure 9 shows an annual time series of simulated/predicted temperature and precipitation in the LFB along with the regression and polynomial lines. The linear and polynomial lines, with strong *R*^2^, showed a definite increase in temperature (Fig. 9a), and linear and polynomial lines fitted to precipitation indicated a decline, with cyclic behavior, showing wet and dry spells in the basin (Fig. 9b). Figure 9a also shows that the temperature range in the basin has reduced over time. This means that relatively cold regions (eastern region) are warming more rapidly than the warmer regions in the basin. In comparison with the other three basins (i.e., CLRB, KYRB, and YENG), the LFB will be warmer than other basins in both FD and SD, and a reduction in precipitation will also be higher than other basin in the FD. However, in the SD, the highest reduction in precipitation was predicted in the Komadugu-Yobe and YENG basins.

**Table 5:** Temperature and precipitation changes in 2016–2025 and 2026–2035 relative to the baseline period (1961–1990) and in the 2020 s relative to the present decade (2006–2015) in the subbasins of the Lake Fitri basin.

Temperature (°C)	East Region	Middle Region	West Region	Basin Mean
Absolute CRU (1961–1990)	26.90	28.50	28.20	27.87
FD (2016–2025)	1.40	1.30	1.00	1.24
SD (2026–2035)	1.90	1.70	1.30	1.60
Absolute CRU 2006–2015	28.28	29.65	29.00	28.98
2020 s (2021–2030)	0.29	0.32	0.41	0.34
**Precipitation**				
Absolute CRU 1961–1990 (mm)	481	517	584	528
FD (2016–2025) (%)	−20	−21	−21	−21
SD (2026–2035) (%)	−4	−2.7	−16	−8
Absolute CRU 2006–2015 (mm)	580	637	619	612
2020s (2021–2030) (%)	−32	−29	−37	−33

FD *First decade*, SD *Second decade*, CRU *Climate Research Unit*.

## Discussion

### Temperature and precipitation trends

In the whole study area, temperature showed an extremely strong increasing trend, with an average rate of 0.022 °C yr^−1^ (1.43 °C 65 yr^−1^), which is even much higher than the global average temperature rate of 0.0074 °C yr^−1^ (0.74 °C 100 yr^−1^)^37^. Sarr^42^ and Funk *et al*.^37^ also examined the faster rate of increasing temperature (approximately 0.023 °C yr^−1^) than the global warming in the West Africa and Chad, respectively, and Collins^68^, over Africa, with an increasing rate of 0.016 °C yr^−1^ C. This mean that if the temperature follows the same rate in the basin, then in the next 100 years, this region may face approximately 2–3 °C hotter climate relative to the current condition. A similar increase in temperature (2.6–4.8 °C) has been reported by IPCC^4^ for the globe, 3–4 °C rise by Collins^68^ over Africa, and 1.6–6 °C increase by Sylla *et al*.^44^ over West Africa at the end of the 21^st^ century. Extremely strong signals of increasing temperature found in this study suggests that the LCB is highly vulnerable to global warming. According to the Fifth Assessment Report of the IPCC, the increasing concentrations of greenhouse gases in the atmosphere has been the dominant cause of observed warming since 1950, with 95% confidence^4^. Africa is the most affected continent due to the global warming, although it contributes least to the global warming, with an average emission of greenhouse gases of 1 metric ton^69^. This increasing temperature in the basin can cause stress on water resources, a reduction in crop productivity, reduction in fishery, more floods and droughts, and increase in vector- and waterborne diseases. However, the LCB already faced a devastating droughts during the 1970s and 1980s, when the lake not only faced high shrinkage but also split into two parts^13^.

This study showed that annual and seasonal precipitations have reduced in the whole study area for the period of the 1951–2015, with moderate downward trends obtained using CRU precipitation and relatively strong downward trends calculated by observed precipitation. Similar results have been explored by Niel *et al*.^70^ in the LCB, Nkiaka *et al*.^38^ in the Logone River basin of the LCB, Funk *et al*.^37^ in Chad, Ifabiyi and Ojoye^71^ over the Sudano-Sahelian Ecological Zone of Nigeria, Sarr^42^ over West Africa, *Conway et al*.^53^ over sub-Saharan Africa regions, and Ali and Lebel^72^ over the Sahel region, with study periods starting before or from 1960s. The study by Niel *et al*.^70^ in the LCB used those stations which are located around LC and not covering the whole study area spatially. These decreasing trends in the LCB could be, to the great extent, due to the Sahel droughts in the 1970s and 1980s, which resulted in devastating impacts on the local population, biodiversity, agriculture, and pastoral activities^38,54^. Sarr^42^ attributed the decreasing trends to a significant drop in rainy days and a slight increase in dry spells during 1960–1990, as well as to the decline in precipitation intensity in the region. Epule *et al*.^73^ reviewed comprehensively the causes and effects of the Sahel droughts and explored the four major possible causes of decreased precipitation in the region: human-induced climate change, dust feedbacks, changes in sea surface temperature, and land and vegetation degradation. Among them, anthropogenic climate change is considered the most influencing factor because it also controls the other three factors. They also revealed some serious implications of the decrease in precipitation on forest and food in the region, especially in the case of agriculture, which provides livelihood to approximately 60% of the population in the LCB^48^ and 80% in most African countries^74^. Dilley *et al*.^75^ revealed that severe food shortages were recorded during the 1982 drought in 27 Sahelian countries with famines in Chad, Sudan, and Ethiopia. On the other hand, Dickson and Steve^76^ reported a decrease in per capita food and drop in food self-sufficiency ratio from 98% in the 1960s to 86% in the mid-1980s due to decreasing precipitation in the region.

However, some studies such as Okonkwo *et al*.^39^, Adeyeri *et al*.^36^, Ndehedehe *et al*.^77^ and Zhu *et al*.^78^ showed an increase in the precipitation in the LCB, and other studies such as Sylla *et al*.^44^, Nicholson^79^, Ibrahim *et al*.^80^, and Mahé and Paturel^81^ also reported, to some extent, similar kind of findings around the LCB, especially over the Sahel belt, but these studies used a study period later than 1980s. It can also be observed in Figs 3 and 4 that precipitation started increasing after the 1980s, especially in the case of annual amounts and the wet season. Another study by Lebel and Ali^82^ indicated that precipitation over the central Sahel increased by approximately 10% for the period of 1990–2007 relative to 1970–1989. Ibrahim *et al*.^80^ have explored not only the increase in the total precipitation but also the increase in the number of rainy days in the last two decades, resulting in the partial recovery of precipitation. This increase could be due to the partial recovery of precipitation during the 1990s in the region, which can be the result of warming over the northern Atlantic Ocean, which draws rains during the wet season in the region^36^. In addition, this increase can be attributed to the increased anthropogenic greenhouse gases and aerosol emission in the atmosphere^54^. Although an increase in precipitation, in general, could have beneficial impacts on agriculture, biodiversity, and pastoral activities, it could also create negative socioeconomic problems, in the form of floods. Floods could also accelerate the risk of water-related diseases where better flood management systems are not present, especially in poor countries. Therefore, the studies with the study period starting before 1980s to the present showed a decrease in precipitation, and the studies with the study period starting after 1980s displayed an increase in precipitation. Spatial analysis of temperature and precipitation is very important to identify the regions at high risk and vulnerable to floods and droughts and to enable local authorities to pay attention on these regions by developing more suitable water management practices and measures.

Precipitation trends obtained by observed data showed a stronger decrease than obtained by the CRU data. This could be because of very limited collected station data (only 8 stations) and cover a very small part of the LCB. The main dataset (CRU) used in the present study also has some uncertainties because this dataset is prepared by interpolating available observed data in the region, which is so scarce. Bastola and François^83^, who evaluated different satellite and the CRU precipitation with gauge data in the Lake LCB, concluded that these datasets overestimate rainfall in the northern semiarid regions of the basin and underestimate it in the humid regions located in the south. They also reported that the number of rain gauges used in the derivation of the CRU dataset has declined markedly from 150 to 75 since 1990 due to financial problem. For further studies, it is suggested to include all stations available in the basin and other global datasets such as GPCC and NCEP to use different trend analysis techniques and to use different time periods to take care of all possible uncertainties. The satellite datasets can also be of good option for the recent trends since the 1990s.

### Temperature and precipitations predictions

This present study suggests that temperature in the basin will increase in the next two decades with respect to the baseline period (1961–1990). The results are well supported by Sylla *et al*.^44^, who used the RCM multimodel approach to project temperature over the Sahel region, under RCP4.5 and RCP8.5. They predicted an average increase of 1.8 °C until 2035 relative to 1976–2005. According to IPCC^84^, global warming would be more intense in Africa. Different studies such as Buontempo^40^, Francois *et al*.^41^, Niang *et al*.^85^, Sarr^42^, *and* Serdeczny *et al*.^10^ have projected an increase in temperature in different parts of Africa, especially over the Sahel.

In some parts of the CLRB, temperature was predicted to decrease in the 2020 s with respect to the present decade (2006–2015). This might be due to the frequent occurrence of forests in these regions^8^. These regions are also included in highly protected natural resource areas in the basin^50^. This result also may be due to assessing changes against a very short period and very recent period (2006–2015). However, we did not find any study showing a decrease in temperature in the future in the region because most studies show temperature anomalies relative to the long-term baseline period (e.g., 1961–1990 or 1971–2000) and not with the very short and recent period that we used. Therefore, further study is needed in the region using the most advanced techniques and tools, such as GCMs and RCMs, with multimodel approaches to confirm these results.

In the case of precipitation, according to the results, the LCB will receive less precipitation in the next two decades relative to the baseline period (1961–1990). This means that in the next 20 years, the basin is going to face more droughts. According to the results, these droughts could be stronger than in the 1970s and 1980s because our baseline period also includes the driest period (1970–1990). Sultan *et al*.^43^ projected a decrease in annual precipitation in the Western Sahel but an increase in the Central Sahel during 2031–2060 under RCP8.5. Vizy *et al*.^46^ showed an increase in summer precipitation during 1941–1960 over the Sahel region, using RCM under RCP8.5, and using RCMs, Sylla *et al*.^45^ projected, for the last two decades of the 21^st^ century, drier conditions over the Sahel region and wetter conditions over the orographic regions According to Niang *et al*.^85^, annual precipitation is *likely* to increase in Central and eastern Africa in the beginning of the mid-21^st^ century, while it is *very likely* to decrease over the Mediterranean areas of northern Africa in the mid and late 21^st^ century with respect to 1986–2005 under RCP8.5. If we examine Figs 6, 7b, 8b and 9b, all figures show an increase in precipitation at the end of 2035 and display a wet and dry cycle, repeating in 20–25 years. Therefore, if we follow this cycle, then after 2035, a wet period may occur for 2035–2060. The above-mentioned studies also showed an increase in precipitation between 2031 and 2060 in the Sahel region, covering the LCB. However, more uncertainties are associated with precipitation projections than temperature projections^86^, and precipitation projections exhibit higher seasonal and spatial dependence than projections of temperature^87^. Future projection of precipitation are quite uncertain because some models predict a significance decrease, some a significant increase, and others no significance change^42^. Although more uncertainty lies in predicting precipitation with time series analysis because precipitation is a heterogeneous variable, the uncertainty cannot be removed completely even by using the most advanced tools such as RCMs and GCMs. Nonetheless, time series analyses are very fast, require less computation, and perform reasonably well.

## Conclusions

In the present study, first, a combination of linear regression, polynomial model, Sens’ slope estimator, and Mann-Kendal test was used to explore temporospatial trends and climate variability in the most active part of the LCB (i.e., the Chari-Logone, Komadugu-Yobe, YENG, and Lake Fitri basins), using observed and CRU annual temperature and precipitation for the period of 1951–2015. Second, temperature and precipitation were predicted by a combination of spectral analysis and harmonic regression for the next 20 years (2016–2035), and decadal changes (2016–2025 and 2026–2035) were obtained with respect to the baseline period (1960–1990) and changes in the 2020 s (2021–2030) with respect to the present decade (PD) (2006–2015). The following are the key findings obtained from the present study:

- Temperature has increased in the study area, with an increasing rate of 0.22 °C decade^−1^. Most increase in temperature was observed in the LFB.
- Annual precipitation has decreased in the study area, with a decreasing rate of 14 mm decade^−1^. Nonetheless, most time series showed weak and nonsignificant decreasing trends. In the CLRB, which contributes 90% to Lake Chad, decreasing trends were even stronger than for the other basins.
- Temperature was predicted to increase in the Lake Chad basin by 0.65 °C and 1.6 °C in the first decade—FD (2016–2025) and second decade—SD (2026–2035), respectively, with respect to 1960–1990. However, unexpectedly, in the CLRB in the 2020 s, temperature was predicted to decrease relative to the PD in some parts of the CLRB, showing cooler climate in the 2020 s. This result might be because these regions are located in highly protected natural resources areas and have dense forests.
- Precipitation was predicted to decrease in all basin, with 4–21% decrease in the FD and 5–16% in the SD relative to 1961–1990. In the 2020 s, precipitation will be decreased by 14–33% relative to the PD in the study area.
- Cyclic behavior was explored in temperature, repeating every 45–49 years, but only in the CLRB, and a cycle of wet and dry periods, repeating 20–25 years, was explored in precipitation in all basins.
- These findings clearly indicate that the Lake Chad basin is highly sensitive to perturbed natural climate, and thus, the water resources of the basin could be highly vulnerable to climate change.

The key findings of the present study may be helpful to water resource managers for planning and managing water resources on a seasonal and annual basis and to policymakers for advising some suitable adaptation and mitigation policies to cope with anticipated climate variability and climate change. However, further studies should include all available observed station data, different trend analysis techniques, different time steps (e.g., monthly and decadal), and should consider a study period before and after the major droughts (e.g., the 1970s) in the Lake Chad basin to cover possible uncertainties. On the other hand, the most advanced tools, such as RCMs/GCMs and multimodel and multi scenarios approaches, could be useful to take care of uncertainties related to prediction of temperature and precipitation. In addition, further study can be done using different time series techniques to predict monthly and seasonal temperature and precipitation, which would be useful for farmers. Furthermore, a standardized precipitation index (SPI) and other relevant indices can be used to monitor flood and droughts in the Lake Chad basin, especially in the Chari-Logone River basin.

## References

[1] Mahmood R, Babel MS (2013). Evaluation of SDSM developed by annual and monthly sub-models for downscaling temperature and precipitation in the Jhelum basin, Pakistan and India. Theor Appl Climatol.

[2] Huang J (2010). Estimation of future precipitation change in the Yangtze River basin by using statistical downscaling method. Stoch. Environ. Res. Risk Assess..

[3] Chu J, Xia J, Xu CY, Singh V (2010). Statistical downscaling of daily mean temperature, pan evaporation and precipitation for climate change scenarios in Haihe River, China. Theor Appl Climatol.

[4] IPCC. Climate Change 2013: The Physical Science Basis. Contribution of Working Group I to the Fifth Assessment Report of the Intergovern-mental Panel on Climate Change 1535 (Cambridge University Press, Cambridge, United Kingdom and New York, NY, USA, 2013).

[5] Syed TH, Famiglietti JS, Chambers DP, Willis JK, Hilburn K (2010). Satellite-based global-ocean mass balance estimates of interannual variability and emerging trends in continental freshwater discharge. Proceedings of the National Academy of Sciences.

[6] Khattak MS, Babel MS, Sharif M (2011). Hydro-meteorological trends in the upper Indus River basin in Pakistan. Clim Res..

[7] IPCC. Summary for policymakers. In: Climate change 2014: Impacts, adaptation, and vulnerability. Part A: Global and sectoral aspects. Contribution of Working Group II to the Fifth Assessment Report of the Intergovermental Panel on Climate Change 1–32 (Cambridge, UK and New York, 2014).

[8] Mahmood R, Jia S (2018). Analysis of causes of decreasing inflow to the Lake Chad due to climate variability and human activities. Hydrol. Earth Syst. Sci. Discuss..

[9] UNFCCC. Climate change: impacts, vulnerabilities and adaptation in developing countries. 68 (Bonn, Germany, 2010).

[10] Serdeczny O (2017). Climate change impacts in Sub-Saharan Africa: from physical changes to their social repercussions. Regional Environmental Change.

[11] Coe MT, Foley JA (2001). Human and natural impacts on the water resources of the Lake Chad basin. J. Geophys. Res-Atmos..

[12] Gao H, Bohn TJ, Podest E, McDonald KC, Lettenmaier DP (2011). On the causes of the shrinking of Lake Chad. Environ. Res. Lett..

[13] Lemoalle J, Bader J-C, Leblanc M, Sedick A (2012). Recent changes in Lake Chad: Observations, simulations and management options (1973–2011). Global Planet. Change.

[14] Tarbuck, E. J. & Lutgens, F. K. In Earth Science (Pearson, 2016).

[15] Feng G (2016). Trend analysis and forecast of precipitation, reference evapotranspiration, and rainfall deficit in the Blackland Prairie of Eastern Mississippi. J. Appl. Meteorol. Clim..

[16] Mahmood R, Jia S (2017). Spatial and temporal hydro-climatic trends in the transboundary Jhelum River basin. Journal of Water and Climate Change.

[17] Nury, A. H., Koch, M. & Alam, M. J. B. In 4th International Conference on Environmental Aspects of Bangladesh. 4 (BENJapan).

[18] Sunday RKM, Masih I, Werner M, van der Zaag P (2014). Streamflow forecasting for operational water management in the Incomati River Basin, Southern Africa. Physics and Chemistry of the Earth, Parts A/B/C.

[19] Teresa R (2015). Using Time Series Analysis to support the Water Resources Management in the Upper Basin of the Suquía River. Pinnacle Environmental & Earth Science.

[20] Esterby, S. R. Review of methods for the detection and estimation of trends with emphasis on water quality applications. *Hydrological Processes* **10**, 127–149, 10.1002/(SICI)1099-1085(199602)10:2<127::AID-HYP354>3.0.CO;2-8 (1996).

[21] Zhang Q, Liu C, Xu C-Y, Xu Y, Jiang T (2006). Observed trends of annual maximum water level and streamflow during past 130 years in the Yangtze River basin, China. J. Hydrol..

[22] Sonali P, Nagesh KD (2013). Review of trend detection methods and their application to detect temperature changes in India. J. Hydrol..

[23] Burn DH, Cunderlik JM, Pietroniro A (2004). Hydrological trends and variability in the Liard River basin / Tendances hydrologiques et variabilité dans le basin de la rivière Liard. Hydrological Sciences Journal.

[24] Fu G, Barber ME, Chen S (2010). Hydro-climatic variability and trends in Washington State for the last 50 years. Hydrological Processes.

[25] Oyerinde Ganiyu Titilope, Hountondji Fabien C. C., Wisser Dominik, Diekkrüger Bernd, Lawin Agnide E., Odofin Ayo J., Afouda Abel (2014). Hydro-climatic changes in the Niger basin and consistency of local perceptions. Regional Environmental Change.

[26] Tekleab S, Mohamed Y, Uhlenbrook S (2013). Hydro-climatic trends in the Abay/Upper Blue Nile basin, Ethiopia. Physics and Chemistry of the Earth, Parts A/B/C.

[27] Wang H (2012). Hydro-climatic trends in the last 50 years in the lower reach of the Shiyang River Basin, NW China. CATENA.

[28] Soltani S, Modarres R, Eslamian SS (2006). The use of time series modeling for the determination of rainfall climates of Iran. International Journal of Climatology.

[29] Yang, L. & Lu, W. In International Symposium on Water Resource and Environmental Protection. 3063–3065 (2011).

[30] Prins, J. In Engineering Statitistics Handbook (NIST/SEMATECH e-Handbook of Statistical Methods) Ch. 6 (2012).

[31] Hill, T., Lewicki, P. & Lewicki, P. Statistics: Methods and Applications: a Comprehensive Reference for Science, Industry, and Data Mining (StatSoft, 2006).

[32] Grzesica D, Więcek P (2016). Advanced Forecasting Methods Based on Spectral Analysis. Procedia. Engineer..

[33] Buttkus, B. Spectral Analysis and Filter Theory in Applied Geophysics. Vol. 1 (Springer, 2000).

[34] Chattopadhyay, A. K. & Chattopadhyay, T. Statistical Methods for Astronomical Data Analysis. Vol. 3 (Springer-Verlag New York, 2014).

[35] Ehrendorfer, M. Spectral Numerical Weather Prediction Models. (Society for Industrial and Applied Mathematics, 2011).

[36] Adeyeri OE, Lamptey BL, Lawin AE, Sanda IS (2017). Spatio-temporal precipitation trend and homogeneity analysis in Komadugu-Yobe basin, Lake Chad region. J. Climatol. Weather Forecasting.

[37] Funk, C. C., Rowland, J., Adoum, A., Eilerts, G. & White, L. A climate trend analysis of Chad. Report No. 2012–3070, (Reston, VA, 2012).

[38] Nkiaka E, Nawaz NR, Lovett JC (2017). Analysis of rainfall variability in the Logone catchment, Lake Chad basin. Int. J. Climatol..

[39] Okonkwo C, Demoz B, Gebremariam S (2014). Characteristics of Lake Chad level variability and links to ENSO, precipitation, and River discharge. Sci. World J..

[40] Buontempo, C. Sahelian climate: part, current, projections. 20 (Met Office Hadley Centre, Devon, United Kingdom, 2010).

[41] Francois E (2015). Projections of rapidly rising surface temperatures over Africa under low mitigation. Environmental Research Letters.

[42] Sarr B (2012). Present and future climate change in the semi-arid region of West Africa: a crucial input for practical adaptation in agriculture. Atmospheric Science Letters.

[43] Sultan B (2014). Robust features of future climate change impacts on sorghum yields in West Africa. Environmental Research Letters.

[44] Sylla, M., Nikiema, M., Gibba, P., Kebe, I. & Klutse, N. A. B. Climate Change over West Africa: Recent Trends and Future Projections (2016).

[45] Sylla, M. B., Gaye, A. T., Jenkins, G. S., Pal, J. S. & Giorgi, F. Consistency of projected drought over the Sahel with changes in the monsoon circulation and extremes in a regional climate model projections. *Journal of Geophysical Research: Atmospheres* **115**, 10.1029/2009JD012983 (2010).

[46] Vizy EK, Cook KH, Crétat J, Neupane N (2013). Projections of a Wetter Sahel in the Twenty-First Century from Global and Regional Models. Journal of Climate.

[47] Buma W, Lee S-I, Seo J (2016). Hydrological evaluation of Lake Chad Basin using space borne and hydrological model observations. Water.

[48] UNEP. Africa’s Lakes: Atlas of our changing environment 89 (Nairobi, Kenya, 2006).

[49] Frenken, K. Irrigation potential in Africa: A basin approach. 177 (1997).

[50] Komble, M. D., Kostoingue, B. & Hamit, A. Report on the state of the Lake Chad Basin ecosystem. 236 (Bonn, Germany, 2016).

[51] Lemoalle, J. & Magrin, G. Le développement du lac Tchad/Development of Lake Chad: Situation actuelle et futurs possibles/Current Situation and Possible Outcomes. 216 (IRD Editions, 2014).

[52] Okonkwo C, Demoz B, Onyeukwu K (2013). Characteristics of drought indices and rainfall in Lake Chad Basin. International Journal of Remote Sensing.

[53] Conway D (2009). Rainfall and Water Resources Variability in Sub-Saharan Africa during the Twentieth Century. Journal of Hydrometeorology.

[54] Dong B, Sutton R (2015). Dominant role of greenhouse-gas forcing in the recovery of Sahel rainfall. Nature Climate Change.

[55] Chiyuan M (2014). Assessment of CMIP5 climate models and projected temperature changes over Northern Eurasia. Environ. Res. Lett..

[56] McMahon T. A., Peel M. C., Karoly D. J. (2015). Assessment of precipitation and temperature data from CMIP3 global climate models for hydrologic simulation. Hydrology and Earth System Sciences.

[57] Smiatek G, Kunstmann H, Knoche R, Marx A (2009). Precipitation and temperature statistics in high-resolution regional climate models: Evaluation for the European Alps. J. Geophys. Res-Atmos..

[58] Harris I, Jones PD, Osborn TJ, Lister DH (2014). Updated high-resolution grids of monthly climatic observations – the CRU TS3.10 Dataset. Int. J. Climatol..

[59] Mann HB (1945). Nonparametric tests against trend. Econometrica.

[60] Kendall, M. G. Rank correlation methods (Charles Griffin). 272 (Oxford University Press, 1975).

[61] Hu Z, Wang L, Wang Z, Hong Y, Zheng H (2015). Quantitative assessment of climate and human impacts on surface water resources in a typical semi-arid watershed in the middle reaches of the Yellow River from 1985 to 2006. Int. J. Climatol..

[62] Sen PK (1968). Estimates of the regression coefficient based on Kendall’s Tau. J. Am. Stat. Assoc..

[63] Kumar S, Merwade V, Kam J, Thurner K (2009). Streamflow trends in Indiana: Effects of long term persistence, precipitation and subsurface drains. J. Hydrol..

[64] Yue S, Pilon P, Phinney B, Cavadias G (2002). The influence of autocorrelation on the ability to detect trend in hydrological series. Hydrological Processes.

[65] Hintze, J. L. User’s guid I: NCSS Statistical Sysstem. 629 (NCSS, Kaysville, Utah, USA, 2007).

[66] Balıbey M, Türkyılmaz S (2015). A time series approach for precipitation in Turkey. Gazi University Journalal of Sciences.

[67] Kozłowski E, Kowalska B, Kowalski D, Mazurkiewicz D (2018). Water demand forecasting by trend and harmonic analysis. Arch. Civ. Mech. Eng..

[68] Collins JM (2011). Temperature Variability over Africa. Journal of Climate.

[69] Fields S (2005). Continental divide: why Africa’s climate change burden is greater. Environ Health Perspect.

[70] Niel H, Leduc C, Dieulin C (2005). Caractérisation de la Variabilité Spatiale et Temporelle des Précipitations Annuelles sur le Bassin du Lac Tchad au Cours du 20ème Siècle/Spatial and Temporal Variability of Annual Rainfall in the Lake Chad Basin During the 20th Century. Hydrological Sciences Journal.

[71] Ifabiyi, I. P. & Ojoye, S. Rainfall Trends in the Sudano-Sahelian Ecological Zone of Nigeria. **2**, 194–202, 10.5539/esr.v2n2p194 (2013).

[72] Ali A, Lebel T (2008). The Sahelian standardized rainfall index revisited. International Journal of Climatology.

[73] Epule TE, Peng C, Lepage L, Chen Z (2014). The causes, effects and challenges of Sahelian droughts: a critical review. Regional Environmental Change.

[74] Epule, T. E., Peng, C., Lepage, L. & Chen, Z. Rainfall and Deforestation Dilemma for Cereal Production in the Sudano-Sahel of Cameroon. *Journal of Agricultural Science* **4**, 10.5539/jas.v4n2p1 (2012).

[75] Dilley Maxx, Chen Robert S., Deichmann Uwe, Lerner-Lam Arthur L., Arnold Margaret (2005). Natural Disaster Hotspots.

[76] Dickson MN, Steve W (1997). Household food insecurity in sub‐Saharan Africa: lessons from Kenya. British Food Journal.

[77] Ndehedehe CE, Agutu NO, Okwuashi O, Ferreira VG (2016). Spatio-temporal variability of droughts and terrestrial water storage over Lake Chad Basin using independent component analysis. J. Hydrol..

[78] Zhu Wenbin, Yan Jiabao, Jia Shaofeng (2017). Monitoring Recent Fluctuations of the Southern Pool of Lake Chad Using Multiple Remote Sensing Data: Implications for Water Balance Analysis. Remote Sensing.

[79] Nicholson S (2005). On the question of the “recovery” of the rains in the West African Sahel. Journal of Arid Environments.

[80] Ibrahim B, Karambiri H, Polcher J, Yacouba H, Ribstein P (2014). Changes in rainfall regime over Burkina Faso under the climate change conditions simulated by 5 regional climate models. Climate Dynamics.

[81] Mahé G, Paturel J-E (2009). 1896–2006 Sahelian annual rainfall variability and runoff increase of Sahelian Rivers. Comptes Rendus Geoscience.

[82] Lebel T, Ali A (2009). Recent trends in the Central and Western Sahel rainfall regime (1990–2007). Journal of Hydrology.

[83] Bastola S, François D (2012). Temporal extension of meteorological records for hydrological modelling of Lake Chad Basin (Africa) using satellite rainfall data and reanalysis datasets. Meteorological Applications.

[84] IPCC. Climate Change 2007: Impacts, Adaptation and Vulnerability. Contribution of Working Group II to the Fourth Assessment Report of the Intergovernmental Panel on Climate Change. 976 (Cambridge, UK, 2007).

[85] Niang, I. *et al*. In Climate Change 2014: Impacts, Adaptation, and Vulnerability. Part B: Regional Aspects. Contribution of Working Group II to the Fifth Assessment Report of the Intergovernmental Panel of Climate Change (eds Barros, V. R. *et al*.) Ch. 22, 1199-1265 (Cambridge University Press, 2014).

[86] Rowell DP (2012). Sources of uncertainty in future changes in local precipitation. Climate Dynamics.

[87] Orlowsky B, Seneviratne SI (2012). Global changes in extreme events: regional and seasonal dimension. Climatic Change.

